# Genetic liability between COVID-19 and heart failure: evidence from a bidirectional Mendelian randomization study

**DOI:** 10.1186/s12872-022-02702-w

**Published:** 2022-06-11

**Authors:** Huachen Wang, Zheng Guo, Yulu Zheng, Bing Chen

**Affiliations:** 1 Department of Emergency Medicine, The Second Hospital of Tianjin Medical University, Tianjin, 300211 People’s Republic of China; 2 Centre for Precision Health, School of Medical and Health Sciences, Edith Cowan University, Perth, 6027 Australia

**Keywords:** COVID-19, Heart failure, Single nucleotide polymorphisms, Instrumental variable, Mendelian randomization study

## Abstract

**Background:**

Previous studies have observed inconsistent associations between coronavirus disease 2019 (COVID-19) and heart failure (HF), but these studies were prone to bias based on reverse causality and residual confounding factors. We aimed to investigate genetic liability between COVID-19 and heart failure using a bidirectional Mendelian randomization study.

**Methods:**

The causal relationship between COVID-19 (including COVID-19, hospitalized COVID-19 compared with the general population, and severe COVID-19) and HF are determined by using a bidirectional Mendelian randomization analysis. We drew on summary statistics from the largest HF genome-wide association study (GWAS) meta-analysis on individuals of European ancestry, which included 47,309 HF patients and 930,014 controls. The inverse variance weighted (IVW), an adaption of the Egger regression (MR-Egger), the weighted median, and weighted model were conducted for the Mendelian randomization analysis to estimate a causal effect. To confirm the stability, we performed a “leave-one-out” approach for the sensitivity analysis.

**Results:**

Genetically predicted severe COVID-19 was not significantly associated with the risk of HF [odds ratio (OR), 1.003; 95% confidence interval (CI), 0.969–1.037; *p* = 0.867]. The IVW demonstrated that there was no association between genetically hospitalized COVID-19 infection and HF risk [OR, 1.009; 95% CI, 0.939–1.085; *p* = 0.797]. There was no evidence to support the association between genetically determined COVID-19 and the risk of HF [OR, 1.066; 95% CI, 0.955–1.190; *p* = 0.253]. In addition, genetically predicted HF was also not causally associated with COVID-19 [OR, 1.162; 95% CI, 0.824–1.639; *p* = 0.393]. MR-Egger analysis indicated no evidence of directional pleiotropy.

**Conclusion:**

The current bidirectional Mendelian randomization analysis overcomes the limitations of observational studies. Our findings indicated that there is no causal association between COVID-19 and HF.

**Supplementary Information:**

The online version contains supplementary material available at 10.1186/s12872-022-02702-w.

## Introduction

Coronavirus disease 2019 (COVID-19), caused by severe acute respiratory syndrome coronavirus type 2 (SARS-COV2), emerged in late 2019 and rapidly developed into a global pandemic [1]. The complications of COVID-19 infection appear to be very broad, including acute respiratory distress syndrome, secondary infection, cardiac injury such as heart failure (HF) [2, 3]. A considerable number of case studies have shown that heart damage, such as arrhythmia, cardiac arrest, and HF, are the end-stage events of COVID-19 patients [4–6]. The mortality rate of patients with heart injury is higher than that of patients without heart injury [5–7]. Previous studies have shown that the vast majority of acute heart failure cases (77.9%) occurred in COVID-19-infected patients with no history of heart failure [8]. Another retrospective cohort study demonstrated that 52% of HF patients infected with COVID-19 died and 12% of the patients recovered and were discharged from the hospital [9]. Some researchers have tried to detect the possible association between COVID-19 infection and HF [10, 11]. Cytokine storms, levels of ACE2, and the levels of angiotensin II may be the causes of HF in the COVID-19 infection population [5, 12–14]. Studies based on endomyocardial biopsy and macrophages showed that SARS-COV2 can reside within the heart myocardial tissue; however, it does not prove that SARS-COV2 plays a direct pathological role in HF [15, 16]. Because of the inconsistent associations of COVID-19 with HF, the causal relationship between COVID-19 and HF infection needs to be further explored.

Mendelian randomization (MR) has become an analytic method that effectively probes genetic proxies that are associated with exposures. Moreover, MR plays a crucial role in separating true causal effects from false associations caused by confounding effects and reverse causal bias [17, 18]. If there is a causal relationship between the exposures and the outcomes, the genetic variation affecting the exposures will also affect the outcomes. MR is considered a natural simulation of a randomized controlled trial because it is based on Mendel's second law, which states that alleles of different genes are independently assigned to each other during gametogenesis. Thus, Mendelian randomization analysis are based on the observation that the inheritance of one trait should be independent of the inheritance of other traits [19]. MR has also been used in cardiovascular research, including heart failure, to detect new underlying aetiological mechanisms and to improve our understanding of the current treatment methods [20, 21]. Therefore, we performed bidirectional MR analysis for determining the genetic variability in COVID-19 patients (including COVID-19, hospitalized COVID-19 compared with the general population and severe COVID-19) is causally associated with HF. Understanding the bidirectional relationship between COVID-19 and HF is of significant public health importance about complications management.

## Materials and methods

### Study design

**Fig. 1:**
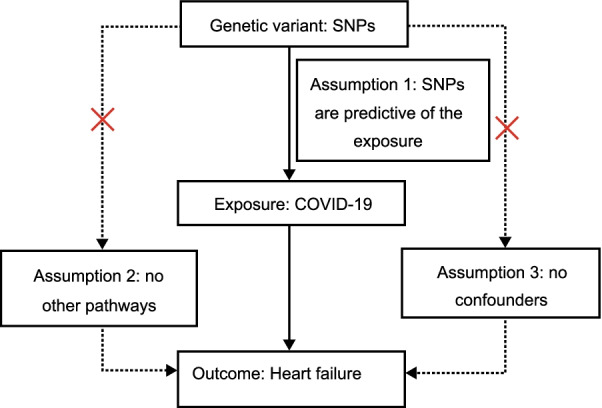
Three significant assumptions of COVID-19 on HF via forward MR. Three different assumptions are represented by three paths. Assumption 1: The SNPs are associated with COVID-19 (the exposure). Assumption 2: The SNPs affect HF only through COVID-19 (exposure) and not via any alternative causal pathways. Assumption 3: The SNPs are completely independent from any potential confounding factors that influence both COVID-19 and HF.

**Fig. 2:**
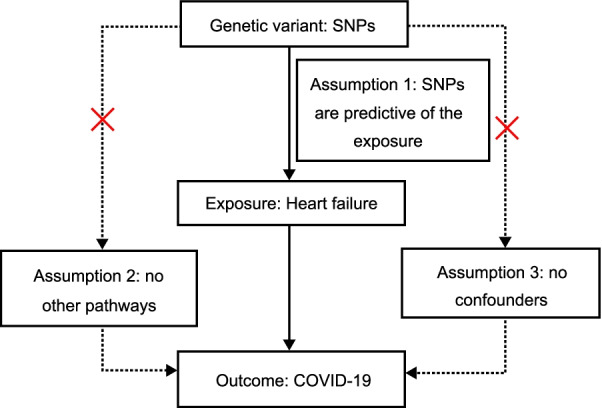
Three significant assumptions of HF with COVID-19 via reverse MR. Three different assumptions are represented by three paths. Assumption 1: The SNPs are associated with HF (the exposure). Assumption 2: The SNPs affect HF only through HF (exposure) and not via any alternative causal pathways. Assumption 3: The SNPs are completely independent from any potential confounding factors that influence both COVID-19 and HF

To ensure a valid MR analysis process, three significant assumptions need to be proven: (1) the SNPs are associated with COVID-19 (HF), (2) the SNPs affect HF (COVID-19) only through COVID-19 (HF) and not via any alternative causal pathways, and (3) the SNPs are completely independent from any potential confounding factors that influence both COVID-19 and HF (Fig. 1, Fig. 2) [22, 23].

### Genetic association datasets for COVID-19

Summary genetic association estimates for the risk of COVID-19 were obtained from the release 5 of COVID-19 GWAS published on January 18, 2021 (https://www.covid19hg.org/results/) [24]. We selected three phenotypes from this GWAS: (1) patients with COVID-19 relative to the general population including 38,984 patients and 1,644,784 control participants; (2) hospitalized patients with COVID-19 compared with the general population including 9986 patients and 1,877,672 control participants; and (3) patients with very severe respiratory-confirmed COVID-19 compared with the general population including 5,101 patients and 1,383,241 control participants. All GWAS summary statistics associated with COVID-19 were based on populations of European ancestry excluding UK Biobank.

### Genetic association datasets for heart failure

We determined the associations between the specific SNPs and HF from the current largest GWAS meta-analysis of HF in populations of European descent [25]. The GWAS meta-analysis, which was conducted by the Heart Failure Molecular Epidemiology for Therapeutic Targets Consortium, included 26 studies (17 population cohort studies, 38,780 HF cases, 893,657 controls and nine case–control studies, 8,529 cases, 36,357 controls), 47,309 patients with heart failure, and 930,014 patients as a control group [25]. This GWAS meta-analysis was adjusted according to sex, age, and principal components. In all of the cohort studies, heart failure was assessed using at least one of the following methods: discharge registration, cause of death registration, or physician decision/diagnosis. Due to insufficient power, the GWAS was not stratified according to the aetiological subtypes.

### Genetic instrumental variables

From the GWAS summary data of COVID-19 patients, we conducted a series of quality control steps to select eligible instrumental SNPs. First, SNPs were chosen as IVs for COVID-19 that were at the threshold from the COVID-19 host genetics initiative. A few significant SNPs of COVID-19 were available using the criterion of *p* < 5 × 10^^−8^. SNPs that achieved significance (p < 5 × 10^^−8^) for HF were selected as IVs. Second, we only extracted the instrumental SNPs for the exposure that were not in linkage disequilibrium ([LD], r^2^ threshold = 0.001). Among those pairs of SNPs, only the SNP with the lowest *P* value was selected. In this study, LD proxies were defined using European samples from the 1000 Genomes Project. To test whether there was a weak bias of the instrumental variable (i.e., genetic variation selected as an instrumental variable was weakly correlated with exposure), we approximated that the F statistic.

$$F\,{\text{statistic}} = \frac{R2 \times (N - 2)}{{(1 - R2)}}$$

$$R^{2} = 2 \times {\text{eaf}} \times \left( {1 - {\text{eaf}}} \right) \times {\text{Beta}}^{2}$$

*N* represents the sample size; eaf represents effect allele frequency.

If the *F* statistic associated with instrument exposure was much greater than 10, then the chance of a weak instrument variable bias was small [26].

### MR analysis

In the primary analysis of the MR analysis, we used the standard inverse-variance weighted (IVW) method to estimate the overall causal relationship between COVID-19 and HF [27]. Using this method, the causal effect of the exposure on the outcome was calculated from the ratio of the SNP associated with the exposures (Wald estimate). According to Mendel's law of inheritance, MR assumes that SNPs are randomly distributed in the general population (separation, independent classification), and this in turn simulates the process of randomization. SNPs always appear before the development of the disease so a reverse causality can be effectively eliminated.

MR is defined as a one-sample MR in a group with complete SNP data, exposure, and results for all participants [20]. Because of the difficulty in the statistics in a single-sample MR, a two-sample MR was developed to enable the analysis of two independent samples: one for focusing on the exposures and the other to focus on the outcomes [22]. In our study, we performed IVW, which is an adaption of Egger regression (MR-Egger), the weighted median approach and the weighted mode approach. These four two-sample MR methods were performed by the "TwoSampleMR" package in R (version 4.0.3) [28, 29]. The related analysis was all one-sided, and evidence of a causal relationship was determined when a prespecified *p*-value was lower than 0.05.

According to the superiorities of each MR, these four methods can complement each other and provide a more plausible causal relationship for our study. In a two-sample MR analysis, we applied the IVW method to analyze the associations between a genetically predicted COVID-19 infection and HF. The MR-Egger method was used to estimate confounding effects and to evaluate directed pleiotropy with weaker assumptions. When 50% or more of the genetic variations were valid instrumental variables, the median-based method could give a reliable effect estimate, which may be more suitable than the MR-Egger method. The weighting method provides a more accurate causal estimation based on a weight analysis. Compared with the traditional MR analysis, the robust method estimates the causal effects that consistently have weaker assumptions.

Traditional IVW methods are the appropriate method to use summary data from a GWAS. We used it to initially estimate the impact between COVID-19 and HF [27]. First, we calculated the IVW average of SNP-specific associations with the fixed effects in HF. In causal reasoning assumptions based on the MR analysis, the analysis will give a biased estimate if one of the tools is invalid [30]. Second, by selecting SNPs that predict COVID-19, we addressed the first hypothesis (the appropriate relationship between SNPs and COVID-19) and the genetic variants that are significant factors for the development of COVID-19 that might satisfy the second hypothesis (without confounding factors). We used MR Egger regression to study the directionality in order to determine any possible violations of the third hypothesis. Subsequently, we created a scatter plot to visually detect the potential pleiotropy by showing the association between each SNP and the risk of HF secondary to COVID-19. The reverse analysis for the effect of HF on COVID-19 is illustrated. A sensitivity analysis explained the potential violations of the sufficient instrumental variable assumption by using MR-Egger regression and the weighted median. Our study also used the “leave-one-out” method for the sensitivity analysis. That is, when the IVW method determined that there was a *P* value < 0.05 and, if the analysis passed the heterogeneity test and the gene diversity test, each related SNP was then removed one by one. The pooled effect of the remaining SNPs was calculated to assess the impact of each SNP.

## Results

To investigate the effect of genetic susceptibility to COVID-19 on HF by utilizing bidirectional MR, we employed independent SNPs that were associated with COVID-19 as instrumental variables. The SNPs that are associated with COVID-19 phenotypes and HF are presented in Additional file 3: Table S1, S2, S3 and S4.

### Causal effect of COVID-19 on HF via forward MR

In the MR analysis, the estimated causal effect between the COVID-19 phenotype and HF is shown in Table 1. There was no genetical association of the severe COVID-19 with HF using 9 SNPs presented in Table 1 (OR, 1.003; 95% CI, 0.969–1.037; *p* = 0.867), without directional pleiotropy (*p* = 0.664) and heterogeneity (*p* = 0.269). By using the IVW method, the genetic predisposition of hospitalized COVID-19 patients compared with the general population was not observed to be statistically significantly associated with HF (OR, 1.009; 95% CI, 0.939–1.085; *p* = 0.797). There was no association between the genetically instrumented COVID-19 and HF risk (OR, 1.066; 95% CI, 0.955–1.190; *p* = 0.253). The MR Egger intercept test further indicated no directional pleiotropy (*p* = 0.207).

**Table 1:** Causal association of COVID-19 with HF via MR analysis

Phenotype	Numbers of SNPs	OR (95% CI)	Beta (SE)	*P*
COVID-19 vs. population				
IVW	5	1.066 (0.955–1.190)	0.064 (0.056)	0.253
Weighted median	5	1.036 (0.912–1.176)	0.035 (0.065)	0.587
Weighted model	5	1.025 (0.855–1.228)	0.024 (0.092)	0.806
MR-Egger	5	0.879 (0.680–1.136)	− 0.129 (0.131)	0.398
egger_intercept			0.011	0.207
*Q* statistic				0.534
Hospitalized COVID-19 vs. population				
IVW	5	1.009 (0.939–1.085)	0.009 (0.037)	0.797
Weighted median	5	1.015 (0.936–1.101)	0.015 (0.041)	0.716
Weighted model	5	1.011 (0.893–1.145)	0.011 (0.063)	0.868
MR-Egger	5	0.825 (0.665–1.024)	− 0.192 (0.110)	0.180
egger_intercept			0.016	0.153
*Q* statistic				0.522
Severe COVID-19 vs. population				
IVW	9	1.003 (0.969–1.037)	0.003 (0.017)	0.867
Weighted median	9	0.987 (0.944–1.031)	− 0.013 (0.022)	0.562
Weighted model	9	0.979 (0.919–1.041)	− 0.022 (0.032)	0.513
MR-Egger	9	0.976 (0.864–1.103)	− 0.024 (0.062)	0.709
egger_intercept			0.014	0.664
*Q* statistic				0.269

*HF* Heart failure, *CI* Confidence interval, *IVW* Inverse-variance weighted; *MR* Mendelian randomization, *MR-Egger* Egger regression, *OR* odds ratio, *SE* Standard error, *SNP* Single-nucleotide polymorphism

Beta is the estimated effect size. *P* < 0.05 was considered statistically significant

**Fig. 3:**
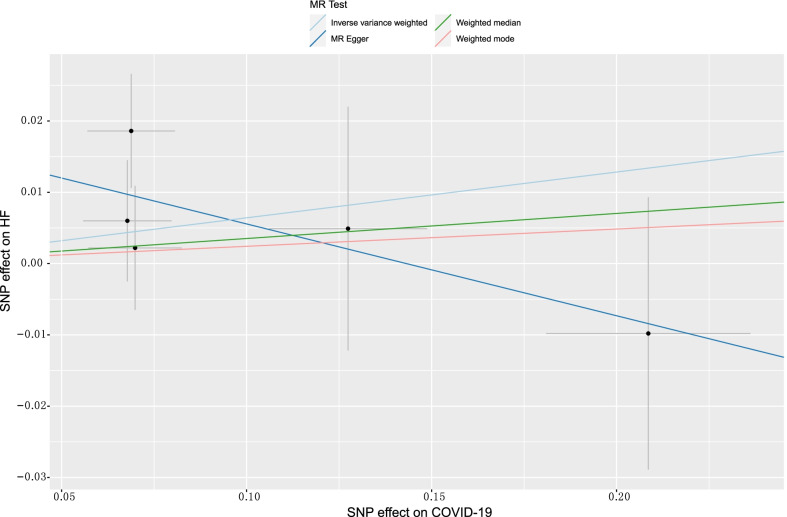
Scatter plot showing the associations of the SNP effects on COVID-19 against the SNP effects on HF. Circles indicate marginal genetic associations with COVID-19 and risk of HF for each variant. Error bars indicate 95% CIs. COVID-19: Coronavirus disease 2019; HF: Heart failure; MR: Mendelian randomization; SNP: Single nucleotide polymorphism

**Fig. 4:**
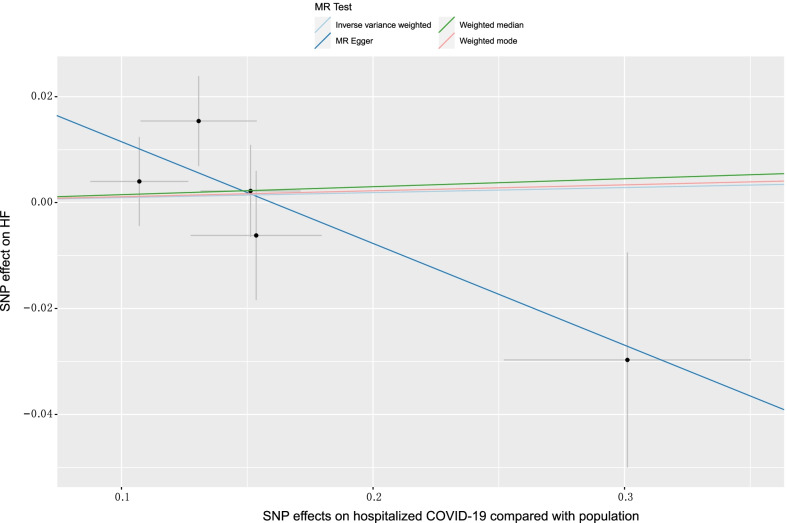
Scatter plot showing the associations of the SNP effects on hospitalized COVID-19 compared with population against the SNP effects on HF. Circles indicate marginal genetic associations with hospitalized COVID-19 and risk of HF for each variant. Error bars indicate 95% CIs. COVID-19: Coronavirus disease 2019; HF: Heart failure; MR: Mendelian randomization; SNP: Single nucleotide polymorphism

**Fig. 5:**
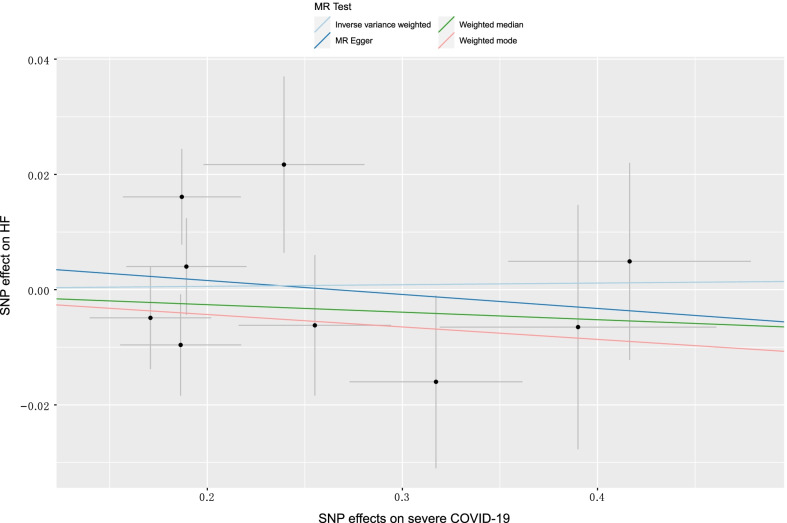
Scatter plot showing the associations of the SNP effects on severe COVID-19 against the SNP effects on HF. Circles indicate marginal genetic associations with severe COVID-19 and the risk of HF for each variant. Error bars indicate 95% CIs. COVID-19: Coronavirus disease 2019; HF: Heart failure; MR: Mendelian randomization; SNP: Single nucleotide polymorphism

In the sensitivity analysis, we observed no consistent causal relationship between genetically predicted COVID-19 phenotypes and HF using the "leave-one-out" approach, and this suggests that there is stability in our results (Additional file 2: Figs. S1–S3). Figures 3, 4 and 5 presented the causal effect of the COVID-19 phenotypes on HF, in which the regression slopes of the lines corresponded to the causal estimates using each of the four different methods.(Additional file 1)

### Causal association of HF with COVID-19 via reverse MR

As shown in Table 2, the reverse MR analysis showed no statistically significant evidence of a causal relationship between HF and COVID-19 (OR, 1.162; 95% CI, 0.824–1.639; *p* = 0.393), hospitalized COVID-19 compared with the general population (OR, 1.173; 95% CI, 0.765–1.780; *p* = 0.464), and severe COVID-19 (OR, 0.539; 95% CI, 0.248–1.173; *p* = 0.119). The relationship between the effect sizes of HF and the phenotypes of COVID-19 are presented in Additional file 2: Figs. S4, S5, and S6. There was no heterogeneity and directional pleiotropy based on the Q test and MR-Egger intercept test. The results of leave-one-out sensitivity analysis showed that the association between genetically instrumented HF with COVID-19 phenotypes were not substantially driven by any individual SNP (Additional file 2: Figs. S7–S9).

**Table 2:** Causal association of HF with COVID-19 via reverse MR analysis

Phenotype	Numbers of SNPs	OR (95% CI)	Beta (SE)	*P*
COVID-19 vs. population				
IVW	4	1.162 (0.824–1.639)	0.150 (0.176)	0.393
Weighted median	4	1.240 (0.912–1.686)	0.215 (0.157)	0.170
Weighted model	4	1.433 (0.850–2.414)	0.360 (0.266)	0.270
MR-Egger	4	0.965 (0.302–3.079)	− 0.036 (0.592)	0.957
egger_intercept			0.034	0.769
*Q* statistic				0.072
Hospitalized COVID-19 vs. population				
IVW	4	1.173 (0.765–1.780)	0.160 (0.218)	0.464
Weighted median	4	1.373 (0.814–2.318)	0.317 (0.267)	0.235
Weighted model	4	1.420 (0.648–3.112)	0.350 (0.400)	0.446
MR-Egger	4	0.564 (0.191–1.669)	− 0.572 (0.553)	0.410
egger_intercept			0.033	0.287
*Q* statistic				0.799
Severe COVID-19 vs. population				
IVW	4	0.539 (0.248–1.173)	− 0.618 (0.396)	0.119
Weighted median	4	0.586 (0.225–1.523)	− 0.535 (0.488)	0.273
Weighted model	4	0.592 (0.198–1.770)	− 0.523 (0.559)	0.418
MR-Egger	4	0.655 (0.060–7.136)	− 0.423 (1.218)	0.762
egger_intercept			0.066	0.881
*Q* statistic				0.859

*HF* Heart failure, *CI* Confidence interval, *IVW* Inverse-variance weighted, *MR* Mendelian randomization, *MR-Egger* Egger regression, *OR* Odds ratio, *SE* Standard error, *SNP* Single-nucleotide polymorphism

Beta is the estimated effect size. *P* < 0.05 was considered statistically significant

## Discussion

To understand the causal relationship between COVID-19 and HF, a bidirectional MR was performed on these two groups using publicly summarized GWAS data. Since genetic variants are substitutes for COVID-19, some other studies have only shown that COVID-19 is associated with HF [28, 29]; however, our study showed no causal effect of COVID-19 genetic liability on the HF risk. Furthermore, there was no MR evidence indicating that genetic liability to HF increases the risk of COVID-19.

Several retrospective studies have shown that HF is the most common clinical manifestation of COVID-19 after acute respiratory distress syndrome (ARDS), respiratory failure, and sepsis [9, 31]. As a possible serious consequence of myocardial injury associated with COVID-19 [32, 33], HF is accompanied by high mortality [13]. Meanwhile, decreased immune function and general weakness in COVID-19 patients may be risk factors for HF. It has been reported that monocytes appear to produce more tumor necrosis factor alpha (TNF-α) and interleukin-10 (IL-10) in COVID-19 patients [34]. TNF-α and IL-10 play different roles in the inflammatory process. TNF-α is one of the most important proinflammatory cytokines. It promotes oxidative stress in areas of inflammation and indirectly causes fever [35]. IL-10 plays a central role in infection by limiting the immune response to pathogens [36].

Therefore, COVID-19 patients may be more prone to myocardial damage and belong to the high-risk group for HF. Although previous observational studies have shown a correlation between COVID-19 and HF, the association has not been established as to whether a genetic susceptibility to COVID-19 plays a causal role in HF. MR studies, an IV-based method to infer the causality between intermediate phenotypes and disease, have been widely conducted in HF research [26].

While a small number of cases directly develop viral-associated myocarditis and this may subsequently lead to HF, in the majority of COVID-19 patients, heart muscle damage or heart failure is due to mechanisms other than natural viral infection alone [15, 32, 37–39]. To date, few cases of COVID-19-related acute myocarditis have been described in the literature. In some cases, SARS-CoV-2 was present in macrophages but not in cardiomyocytes. Furthermore, endomyocardial biopsies have showed only low-grade interstitial myocardial inflammation and specific changes in cardiac myocytes, including myofibrillar lysis and lipid droplets [40]. These findings suggest that the virus can reside in the heart, but these findings do not prove that the virus has a direct pathogenic role in heart failure [15, 16]. A recent study found that HF were not associated with the risk of COVID-19 severity [41]. Our novel results first indicated that the genetic liability of COVID-19 had no causal effects on the risk of HF, which was consistent with previous studies [15, 16]. These findings implied that physicians should treat COVID-19 as a genuine confounder and should pay more attention to the other factors in high-risk individuals.

Our MR research has many advantages. First, we performed four complementary MR methods to prevent a reverse causal bias. Second, various SNPs were used as a tool to study COVID-19, which made it possible to detect the HF risk and provided a powerful genetic tool for COVID-19 infection. Third, the IVs used in our study were independent SNPs that could minimize interference from linkage disequilibrium.

There are also some limitations to our study. First, the HF GWAS is from a pre-COVID-19 pandemic cohort. This cohort does not have HF cases that are related to COVID-19 infection and cannot capture the genetic variants influencing the predisposition to COVID-19-related HF. Future MR studies are needed when a HF GWAS using post-COVID-19 cohort is available. Second, a potential limitation of our study is that some data may overlap across HF. Theoretically, ideal data should be obtained from independent samples. However, in practice, the initial GWAS studies mixed some samples. Therefore, we used strong instruments (i.e., F statistic much greater than 10) to minimize the bias caused by overlapping [29]. Third, the genetic data from the COVID-19 study focused on susceptibility and severity. The susceptibility to and severity of COVID-19 were mixed in the original data. Our work requires more specific clarification of the issue, but at present, we have no methods to distinguish the two classifications. Fourth, the small number of variants associated with COVID-19 may have limited the statistical power in our study. Fifth, although we used multiple methods to rule out pleiotropy, the link between SNPs and COVID-19 may still be through other means. We also cannot exclude a common genetic basis between COVID-19 and HF. These results were only derived from a statistical analysis, so physicians must be more cautious about patients with COVID-19. Thus, although our analysis did not provide evidence of an association between genetic responsibility for COVID-19 and HF, it does not exclude the value of COVID-19 infection in HF risk prediction. In the future, more research is needed to explore the association between COVID-19 and HF from different perspectives.

## Conclusions

This analysis used bidirectional MR to explore potential causal associations between COVID-19 and HF. Although there have been efforts to search for causal mechanisms linking the two diseases, our analysis found that COVID-19 is not causally associated with HF.

## Supplementary Information

**Additional file1:** MR analysis

**Additional file 2: Fig.S1** MRleave-one-outsensitivityanalysisforCOVID-19andHF. MR:mendelianrandomization;COVID-19:CoronavirusDisease2019;HF:heartfailure. **Fig. S2** MRleave-one-outsensitivityanalysisforhospitalizedCOVID-19andHF. MR:mendelianrandomization;COVID-19:CoronavirusDisease2019;HF:heartfailure.** Fig. S3** MRleave-one-outsensitivityanalysisforsevereCOVID-19andHF. MR: mendelianrandomization;COVID-19:CoronavirusDisease2019;HF:heartfailure.** Fig. S4** ScatterplotshowingtheassociationsoftheSNPeffectsonHFagainsttheSNPeffectsonCOVID-19. CirclesindicatemarginalgeneticassociationswithHFandriskofCOVID-19foreachvariant.Errorbarsindicate95%CIs.COVID-19: Coronavirusdisease2019;HF: Heartfailure;MR: Mendelianrandomization;SNP: Singlenucleotidepolymorphism. **Fig. S5 **ScatterplotshowingtheassociationsoftheSNPeffectsonHFcomparedwithpopulationagainsttheSNPeffectsonhospitalizedCOVID-19. CirclesindicatemarginalgeneticassociationswithHFandriskofhospitalizedCOVID-19foreachvariant.Errorbarsindicate95%CIs.COVID-19:Coronavirusdisease2019;HF: Heartfailure;MR: Mendelianrandomization;SNP: Singlenucleotidepolymorphism. **Fig. S6** ScatterplotshowingtheassociationsoftheSNPeffectsonHFagainsttheSNPeffectsonsevereCOVID-19. CirclesindicatemarginalgeneticassociationswithHFandtheriskofsevereCOVID-19foreachvariant.Errorbarsindicate95%CIs.COVID-19: Coronavirusdisease2019;HF: Heartfailure;MR: Mendelianrandomization;SNP: Singlenucleotidepolymorphism. **Fig. S7 **MRleave-one-outsensitivityanalysisforHFandCOVID-19. MR: mendelianrandomization;COVID-19: CoronavirusDisease2019;HF: heartfailure. **Fig. S8 **MRleave-one-outsensitivityanalysisforHFandhospitalizedCOVID-19. MR: mendelianrandomization;COVID-19:CoronavirusDisease2019;HF:heartfailure. **Fig. S9** MRleave-one-outsensitivityanalysisforHFandsevereCOVID-19. MR: mendelianrandomization;COVID-19: CoronavirusDisease2019;HF:heartfailure

**Additional file 3:** **Table S1 **The SNPs that are associated with COVID-19. **Table S2** The SNPs that are associated with hospitalized COVID-19. **Table S3** The SNPs that are associated with severe COVID-19. **Table S4** The SNPs that are associated with heart failure.

## Data Availability

The data used to support the findings of this study are included in the article. The datasets used and/or analysed during the current study are available from the corresponding author on reasonable request.

## Abbreviations

COVID-19: Coronavirus disease 2019
HF: Heart failure
SNPs: Single nucleotide polymorphisms
IVs: Instrumental variables
GWAS: Genome-wide association studies
IVW: Inverse variance weighted
MR-Egger: An adaption of egger regression
SARS-COV2: Severe acute respiratory syndrome coronavirus 2
MR: Mendelian randomization
CIs: Confidence intervals
ORs: Odds ratios
ARDS: Acute respiratory distress syndrome
TNF-*α*: Tumour necrosis factor *α*
IL-10: Interleukin-10
