# Supplementary material for: Genetic liability between COVID-19 and heart failure: evidence from a bidirectional Mendelian randomization study
Source: BMC Cardiovasc Disord. 2022 Jun 11;22:262. doi: 10.1186/s12872-022-02702-w (PMC9188011; doi:10.1186/s12872-022-02702-w)
Supplement: Supplementary file 1 — Additional file1: MR analysis [file 12872_2022_2702_MOESM1_ESM.docx]

**MR analysis**

The primary analysis of the current MR analysis was conducted by the IVW approach, combining the effect estimate of each single IV. Results are presented as odds ratio (OR) and 95% confidence interval (CI). Sensitivity analyses were conducted using MR-Egger regression, weighted median, and weighted mode. These methods hold different assumptions at the costs of reduced statistical power. MR-Egger method provided concordant results even when up to 50% of the genetic variants were invalid. The weighted median allows for 50% of the IVs to be invalid or present pleiotropy. The MR-Egger intercept test was also conducted to test the horizontal pleiotropy. We applied a leave-one-out analysis testing as sensitivity analysing approaches to ensure the robustness of results.
