# Supplementary material for: Genetic liability between COVID-19 and heart failure: evidence from a bidirectional Mendelian randomization study
Source: BMC Cardiovasc Disord. 2022 Jun 11;22:262. doi: 10.1186/s12872-022-02702-w (PMC9188011; doi:10.1186/s12872-022-02702-w)
Supplement: Supplementary file 2 — Additional file 2: Fig.S1 MRleave-one-outsensitivityanalysisforCOVID-19andHF. MR:mendelianrandomization;COVID-19:CoronavirusDisease2019;HF:heartfailure. Fig. S2 MRleave-one-outsensitivityanalysisforhospitalizedCOVID-19andHF. MR:mendelianrandomization;COVID-19:CoronavirusDisease2019;HF:heartfailure. Fig. S3 MRleave-one-outsensitivityanalysisforsevereCOVID-19andHF. MR: mendelianrandomization;COVID-19:CoronavirusDisease2019;HF:heartfailure. Fig. S4 ScatterplotshowingtheassociationsoftheSNPeffectsonHFagainsttheSNPeffectsonCOVID-19. CirclesindicatemarginalgeneticassociationswithHFandriskofCOVID-19foreachvariant.Errorbarsindicate95%CIs.COVID-19: Coronavirusdisease2019;HF: Heartfailure;MR: Mendelianrandomization;SNP: Singlenucleotidepolymorphism. Fig. S5 ScatterplotshowingtheassociationsoftheSNPeffectsonHFcomparedwithpopulationagainsttheSNPeffectsonhospitalizedCOVID-19. CirclesindicatemarginalgeneticassociationswithHFandriskofhospitalizedCOVID-19foreachvariant.Errorbarsindicate95%CIs.COVID-19:Coronavirusdisease2019;HF: Heartfailure;MR: Mendelianrandomization;SNP: Singlenucleotidepolymorphism. Fig. S6 ScatterplotshowingtheassociationsoftheSNPeffectsonHFagainsttheSNPeffectsonsevereCOVID-19. CirclesindicatemarginalgeneticassociationswithHFandtheriskofsevereCOVID-19foreachvariant.Errorbarsindicate95%CIs.COVID-19: Coronavirusdisease2019;HF: Heartfailure;MR: Mendelianrandomization;SNP: Singlenucleotidepolymorphism. Fig. S7 MRleave-one-outsensitivityanalysisforHFandCOVID-19. MR: mendelianrandomization;COVID-19: CoronavirusDisease2019;HF: heartfailure. Fig. S8 MRleave-one-outsensitivityanalysisforHFandhospitalizedCOVID-19. MR: mendelianrandomization;COVID-19:CoronavirusDisease2019;HF:heartfailure. Fig. S9 MRleave-one-outsensitivityanalysisforHFandsevereCOVID-19. MR: mendelianrandomization;COVID-19: CoronavirusDisease2019;HF:heartfailure [file 12872_2022_2702_MOESM2_ESM.pdf]

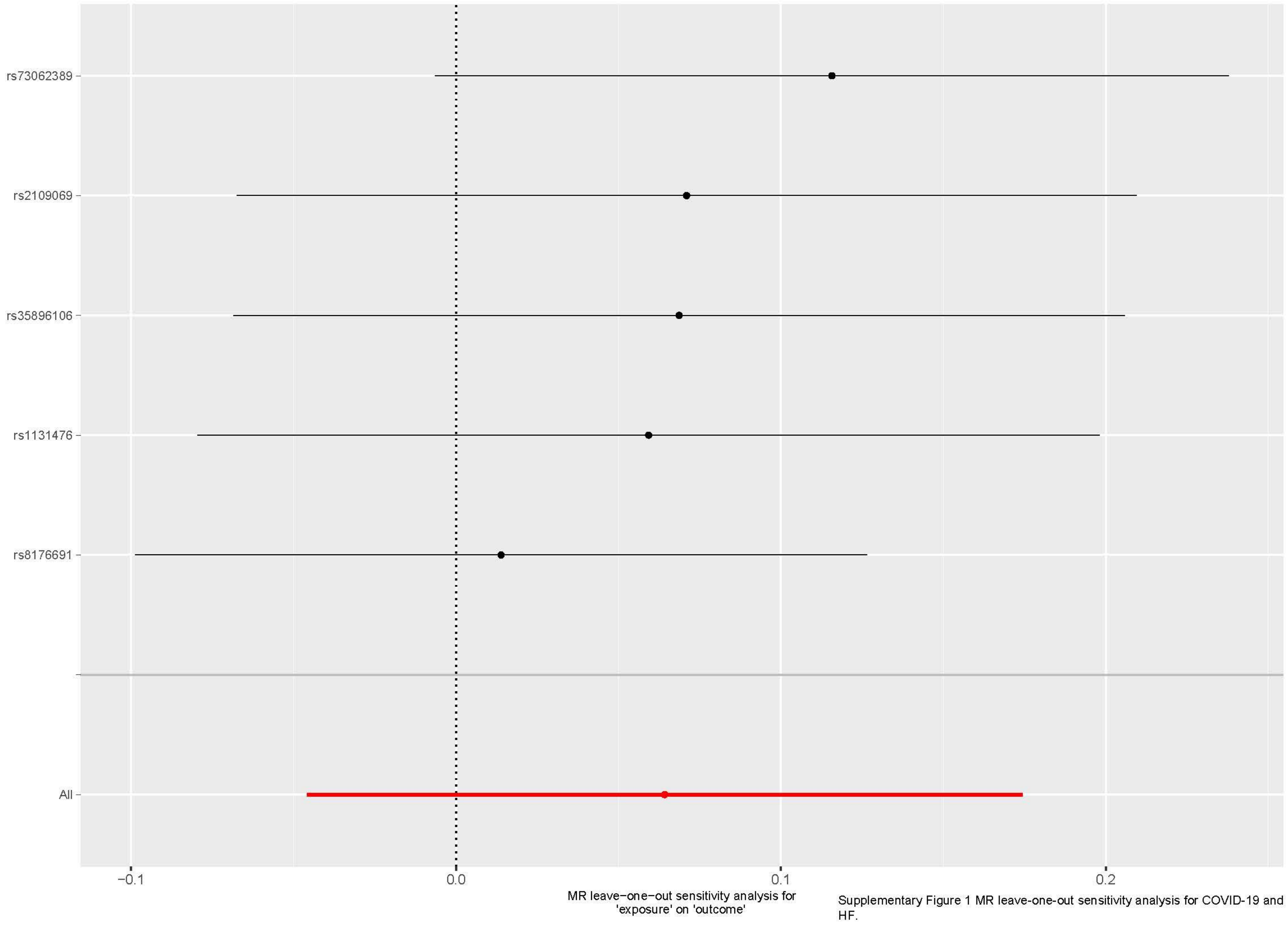

Supplementary Figure 1 MR leave-one-out sensitivity analysis for COVID-19 and HF.

MR: mendelian randomization; COVID-19： Coronavirus Disease 2019； HF： heart failure.

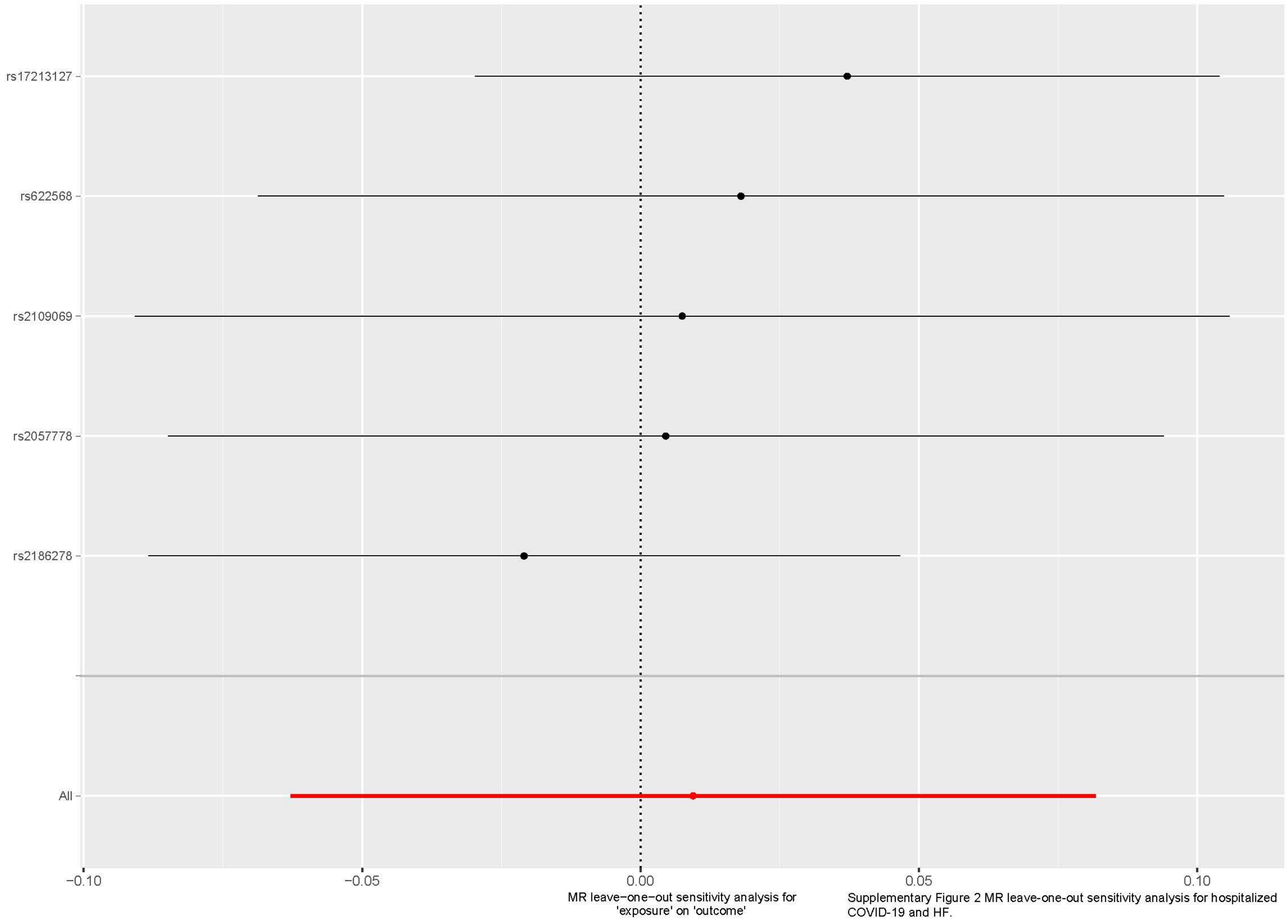

Supplementary Figure 2 MR leave-one-out sensitivity analysis for hospitalized COVID-19 and HF.

MR: mendelian randomization; COVID-19：Coronavirus Disease 2019； HF： heart failure

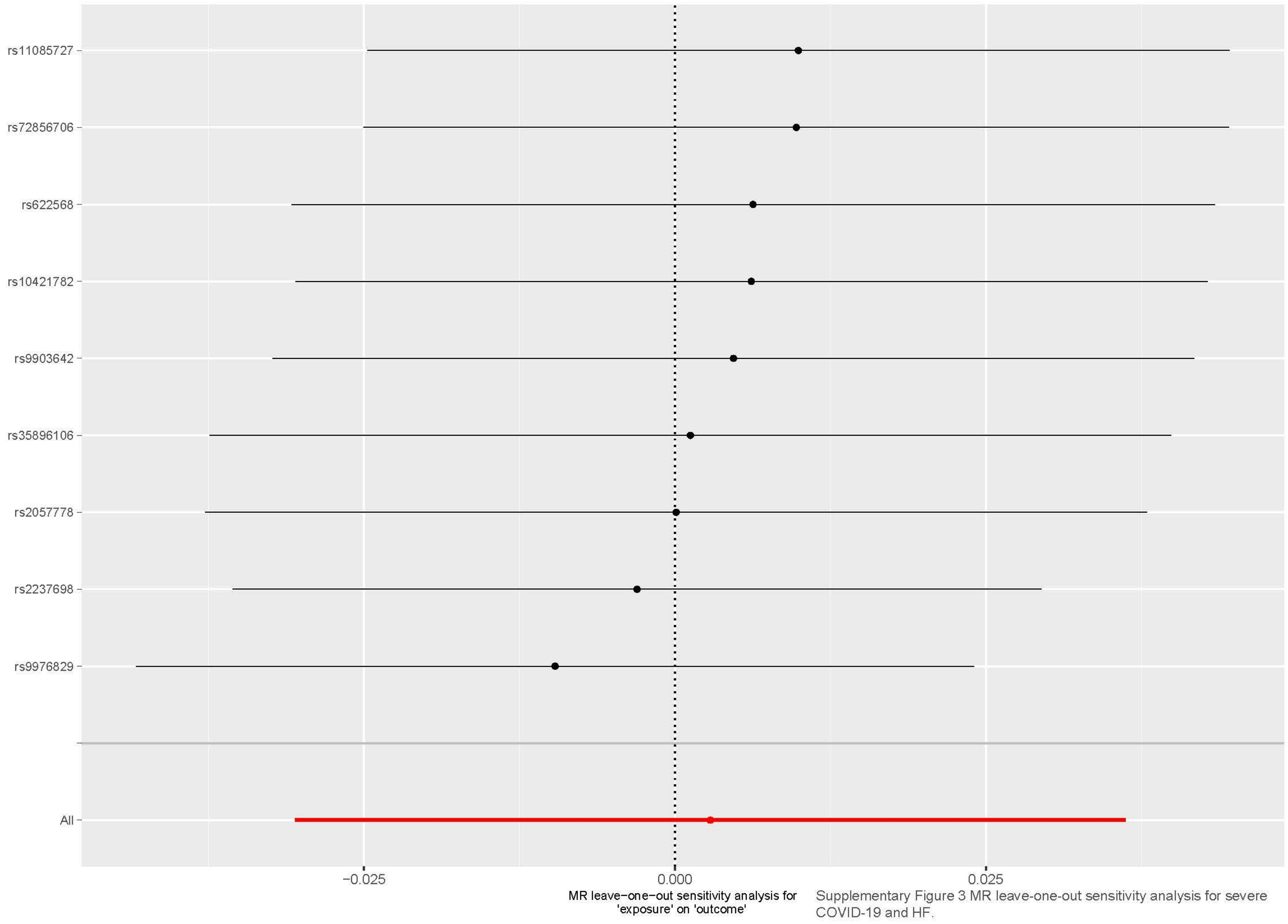

Supplementary Figure 3 MR leave-one-out sensitivity analysis for severe COVID-19 and HF.

MR: mendelian randomization; COVID-19：Coronavirus Disease 2019； HF： heart failure

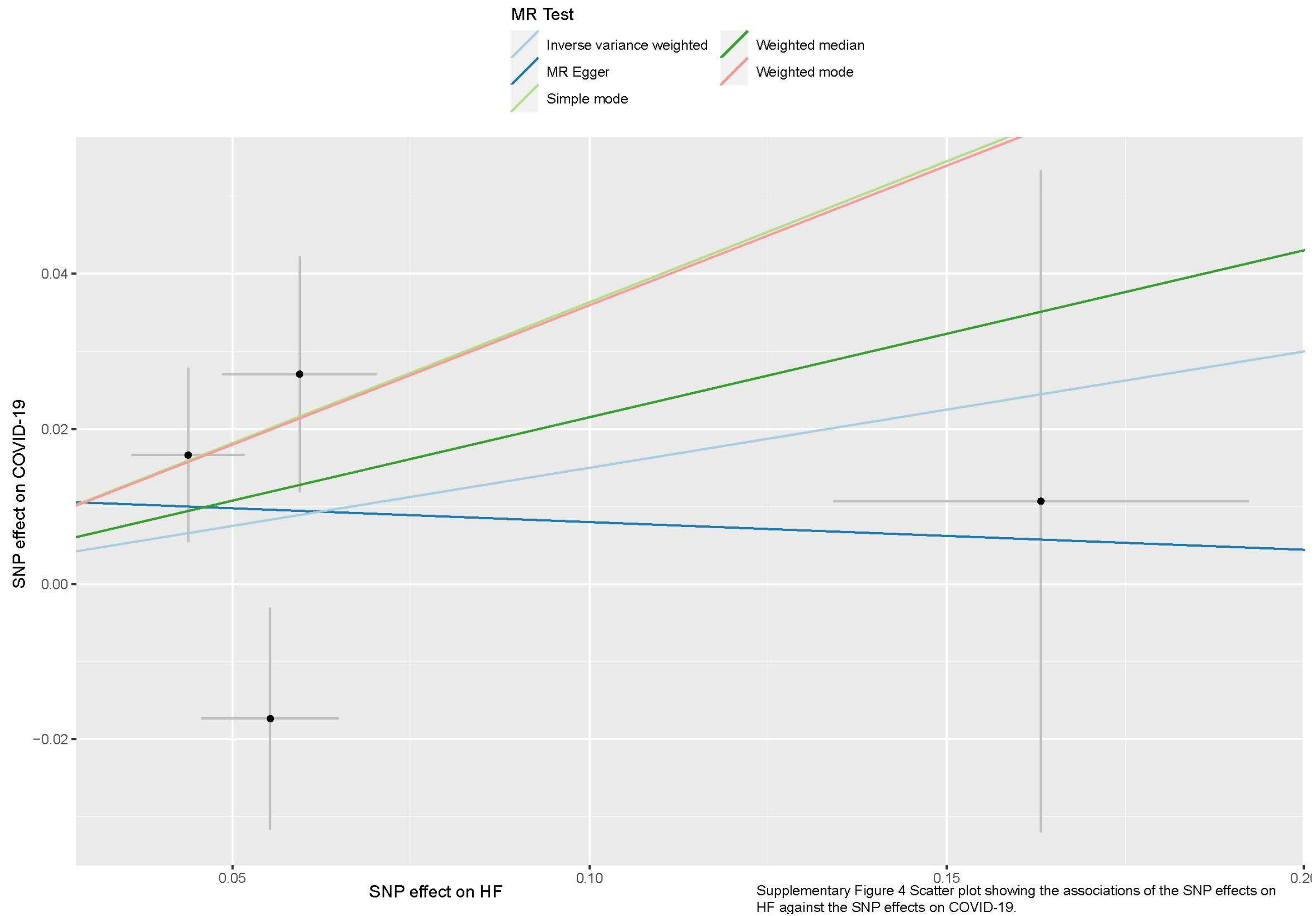

Supplementary Figure 4 Scatter plot showing the associations of the SNP effects on HF against the SNP effects on COVID-19.

Supplementary Figure 4 Scatter plot showing the associations of the SNP effects on HF against the SNP effects on COVID-19.

Circles indicate marginal genetic associations with HF and risk of COVID-19 for each variant. Error bars indicate 95% CIs. COVID-19: Coronavirus disease 2019; HF: Heart failure; MR: Mendelian randomization; SNP: Single nucleotide polymorphism.

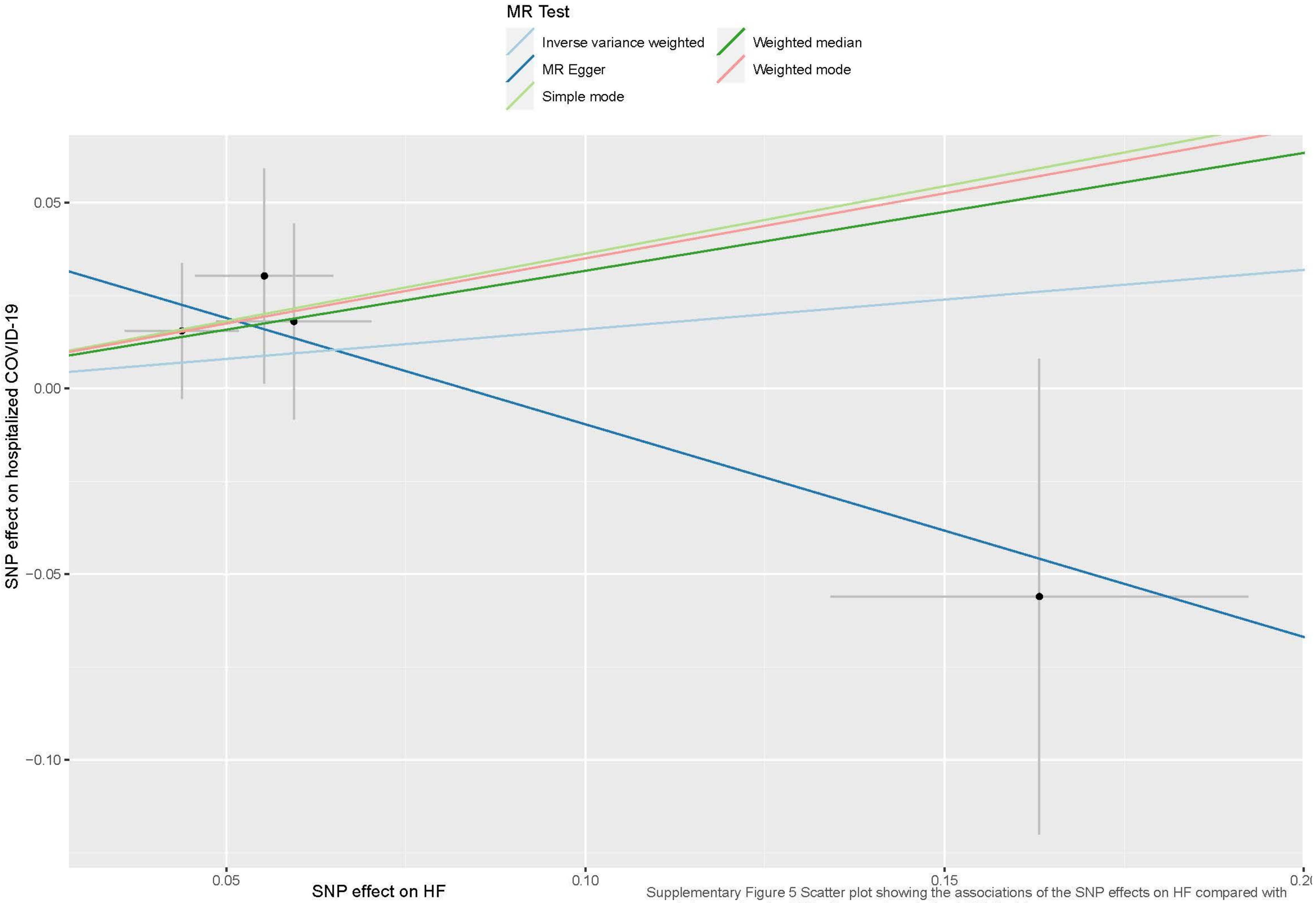

Supplementary Figure 5 Scatter plot showing the associations of the SNP effects on HF compared with population against the SNP effects on hospitalized COVID-19.

Supplementary Figure 5 Scatter plot showing the associations of the SNP effects on HF compared with population against the SNP effects on hospitalized COVID-19.

Circles indicate marginal genetic associations with HF and risk of hospitalized COVID-19 for each variant. Error bars indicate 95% CIs. COVID-19: Coronavirus disease 2019; HF: Heart failure; MR: Mendelian randomization; SNP: Single nucleotide polymorphism.

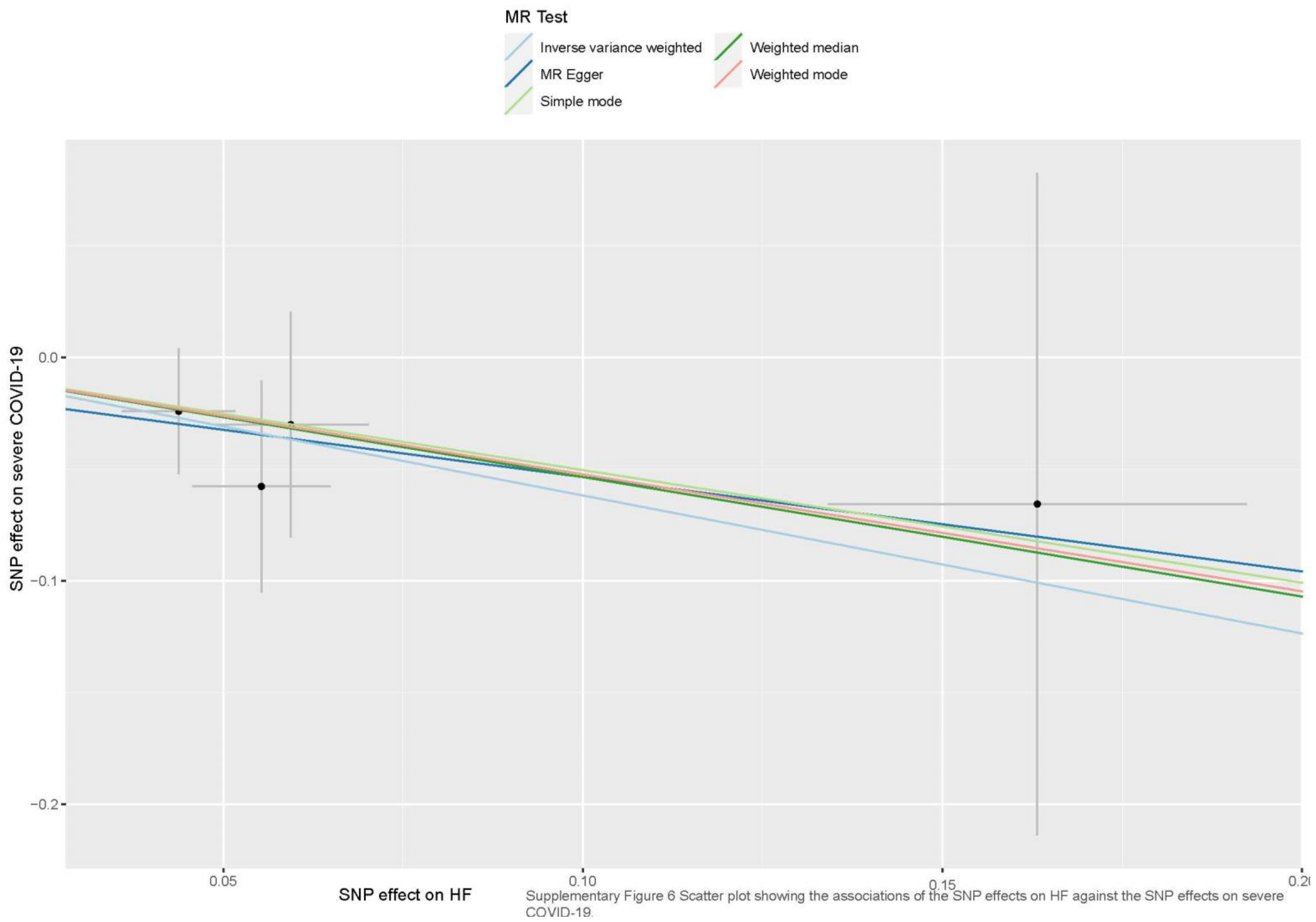

Supplementary Figure 6 Scatter plot showing the associations of the SNP effects on HF against the SNP effects on severe COVID-19.

Circles indicate marginal genetic associations with HF and the risk of severe COVID-19 for each variant. Error bars indicate 95% CIs.

COVID-19: Coronavirus disease 2019; HF: Heart failure; MR: Mendelian randomization; SNP: Single nucleotide polymorphism.

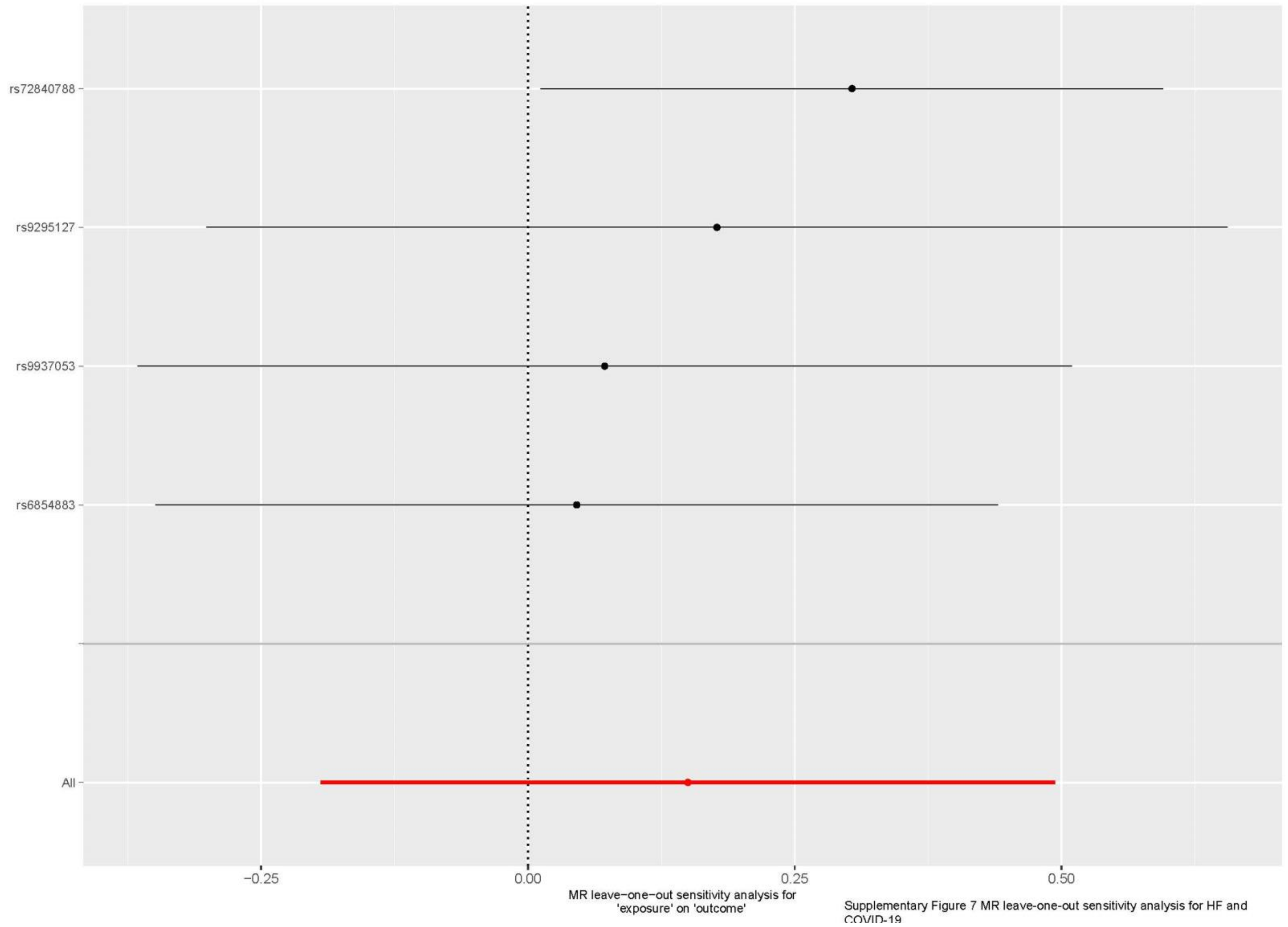

Supplementary Figure 7 MR leave-one-out sensitivity analysis for HF and COVID-19.

MR: mendelian randomization; COVID-19： Coronavirus Disease 2019； HF： heart failure

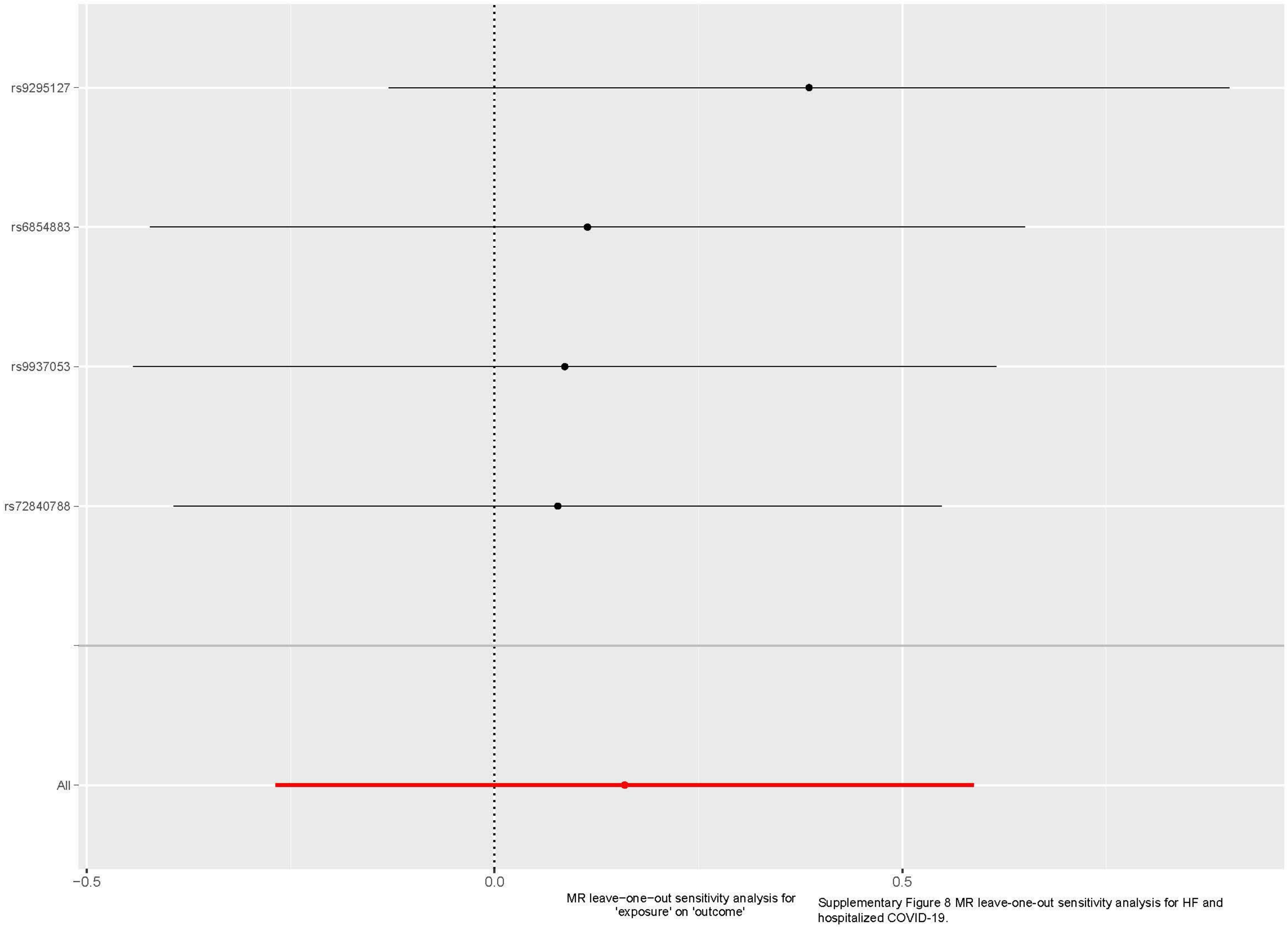

Supplementary Figure 8 MR leave-one-out sensitivity analysis for HF and hospitalized COVID-19.

MR: mendelian randomization; COVID-19： Coronavirus Disease 2019； HF： heart failure

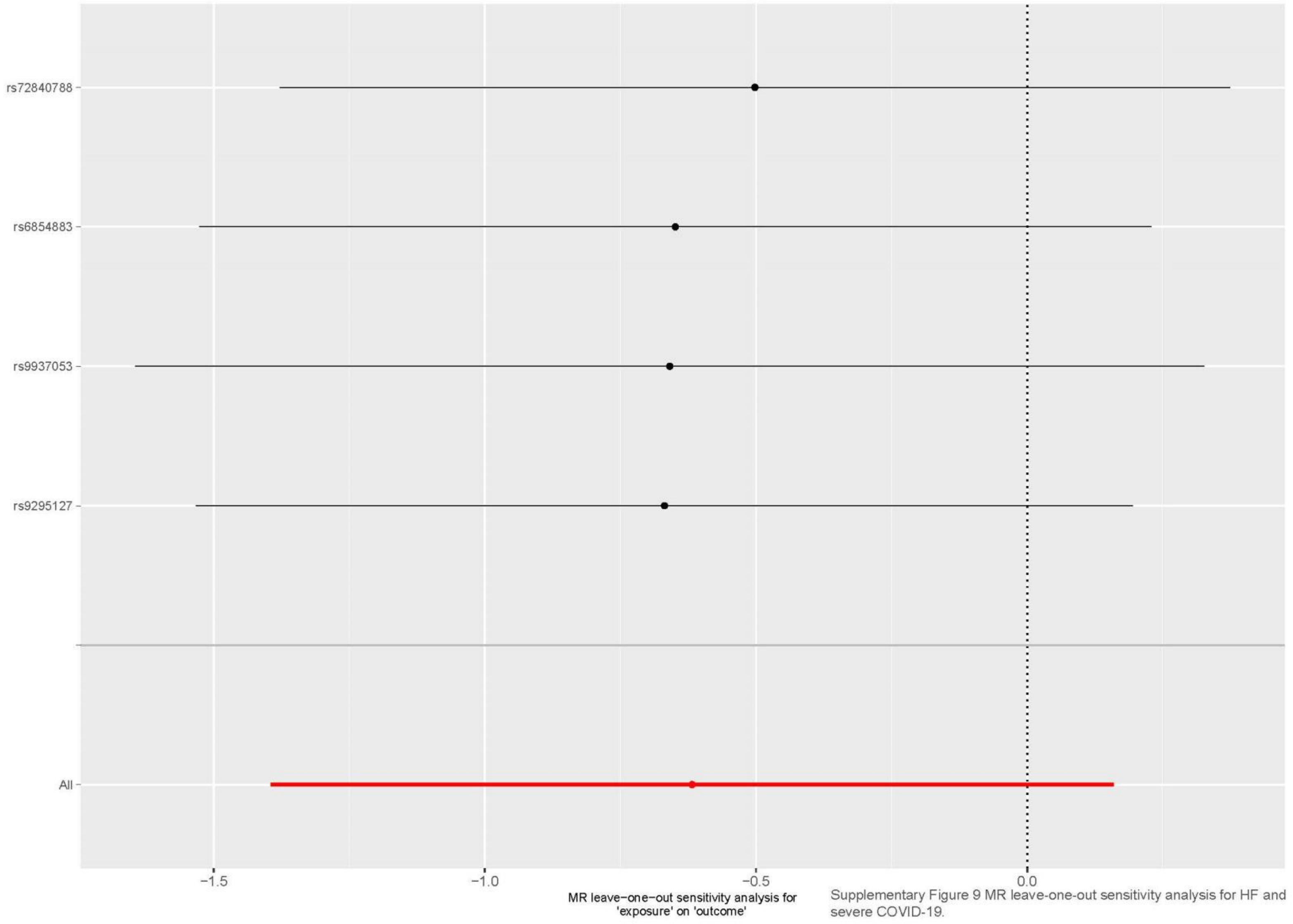

Supplementary Figure 9 MR leave-one-out sensitivity analysis for HF and severe COVID-19.

MR: mendelian randomization; COVID-19： Coronavirus Disease 2019； HF： heart failure
