# Supplementary material for: Genetic liability between COVID-19 and heart failure: evidence from a bidirectional Mendelian randomization study
Source: BMC Cardiovasc Disord. 2022 Jun 11;22:262. doi: 10.1186/s12872-022-02702-w (PMC9188011; doi:10.1186/s12872-022-02702-w)
Supplement: Supplementary file 3 — Additional file 3: Table S1 The SNPs that are associated with COVID-19. Table S2 The SNPs that are associated with hospitalized COVID-19. Table S3 The SNPs that are associated with severe COVID-19. Table S4 The SNPs that are associated with heart failure. [file 12872_2022_2702_MOESM3_ESM.docx]

**Supplementary Table 1.** The SNPs that are associated with COVID-19.

| **SNP** | **Chr.** | **A1** | **A2** | **Beta Estimate** | **SE** | **Discovery *p*** |
| --- | --- | --- | --- | --- | --- | --- |
| rs73062389 | 3 | G | A | 0.20857 | 0.027611 | 4.224E-14 |
| rs35896106 | 3 | C | T | 0.12742 | 0.021382 | 2.534E-09 |
| rs8176691 | 9 | C | T | -0.068815 | 0.011845 | 6.255E-09 |
| rs1131476 | 12 | G | A | 0.067775 | 0.011962 | 1.463E-08 |
| rs2109069 | 19 | G | A | 0.069868 | 0.01261 | 3.012E-08 |

SNP: single nucleotide polymorphism; Chr.: chromosome; A1: effect allele; A2: reference allele; SE: standard error.

**Supplementary Table 2.** The SNPs that are associated with hospitalized COVID-19.

| **SNP** | **Chr.** | **A1** | **A2** | **Beta**  **Estimate** | **SE** | **Discovery *p*** |
| --- | --- | --- | --- | --- | --- | --- |
| rs17213127 | 3 | C | T | 0.30111 | 0.049122 | 8.80E-10 |
| rs622568 | 7 | A | C | 0.15355 | 0.026026 | 3.64E-09 |
| rs2057778 | 12 | G | T | 0.10708 | 0.019478 | 3.85E-08 |
| rs2109069 | 19 | G | A | 0.15131 | 0.019906 | 2.94E-14 |
| rs2186278 | 21 | T | G | 0.13067 | 0.023104 | 1.55E-08 |

SNP: single nucleotide polymorphism; Chr.: chromosome; A1: effect allele; A2: reference allele; SE: standard error.

**Supplementary Table 3.** The SNPs that are associated with severe COVID-19.

| **SNP** | **Chr.** | **A1** | **A2** | **Beta**  **Estimate** | **SE** | **Discovery *p*** |
| --- | --- | --- | --- | --- | --- | --- |
| rs35896106 | 3 | C | T | 0.41646 | 0.062204 | 2.156E-11 |
| rs72856706 | 6 | C | T | 0.31722 | 0.044289 | 7.923E-13 |
| rs622568 | 7 | A | C | 0.25514 | 0.039109 | 6.861E-11 |
| rs2237698 | 7 | C | T | 0.2393 | 0.041252 | 6.593E-09 |
| rs2057778 | 12 | G | T | 0.18928 | 0.030796 | 7.939E-10 |
| rs9903642 | 17 | G | A | 0.39003 | 0.070891 | 3.759E-08 |
| rs10421782 | 19 | A | C | -0.17087 | 0.031136 | 4.066E-08 |
| rs11085727 | 19 | C | T | 0.18635 | 0.031033 | 1.915E-09 |
| rs9976829 | 21 | G | A | -0.18695 | 0.030207 | 6.058E-10 |

SNP: single nucleotide polymorphism; Chr.: chromosome; A1: effect allele; A2: reference allele; SE: standard error.

**Supplementary Table 4.** The SNPs that are associated with heart failure.

| **SNP** | **Chr.** | **A1** | **A2** | **Beta**  **Estimate** | **SE** | **Discovery *p*** |
| --- | --- | --- | --- | --- | --- | --- |
| rs6854883 | 4 | C | T | 5.94E-02 | 0.0108 | 3.67E-08 |
| rs9295127 | 6 | C | A | 1.63E-01 | 0.0291 | 2.00E-08 |
| rs72840788 | 10 | G | A | -5.53E-02 | 0.0096 | 9.06E-09 |
| rs9937053 | 16 | G | A | 0.0438 | 0.0079 | 2.99E-08 |

SNP: single nucleotide polymorphism; Chr.: chromosome; A1: effect allele; A2: reference allele; SE: standard error.

Supplementary Table Legends

Supplementary Table 1. The SNPs that are associated with COVID-19.

SNP: single nucleotide polymorphism; Chr.: chromosome; A1: effect allele; A2: reference allele; SE: standard error.

Supplementary Table 2. The SNPs that are associated with hospitalized COVID-19.

SNP: single nucleotide polymorphism; Chr.: chromosome; A1: effect allele; A2: reference allele; SE: standard error.

Supplementary Table 3. The SNPs that are associated with severe COVID-19.

SNP: single nucleotide polymorphism; Chr.: chromosome; A1: effect allele; A2: reference allele; SE: standard error.

Supplementary Table 4. The SNPs that are associated with heart failure.

SNP: single nucleotide polymorphism; Chr.: chromosome; A1: effect allele; A2: reference allele; SE: standard error.
